# Sero-epidemiology of human coronaviruses in three rural communities in Ghana

**DOI:** 10.11604/pamj.2021.38.244.26110

**Published:** 2021-03-08

**Authors:** Michael Owusu, Augustina Angelina Sylverken, Philip El-Duah, Godfred Acheampong, Mohammed Mutocheluh, Yaw Adu-Sarkodie

**Affiliations:** 1 Department of Medical Diagnostics, Kwame Nkrumah University of Science and Technology, Kumasi, Ghana,; 2 Kumasi Centre for Collaborative Research in Tropical Medicine, Kwame Nkrumah University of Science and Technology, Kumasi, Ghana,; 3 Centre for Health Systems Strengthening, Kumasi, Ghana,; 4 Department of Theoretical and Applied Biology, Kwame Nkrumah University of Science and Technology, Kumasi, Ghana,; 5 Institute of Virology, Charite, Universitätsmedizin Berlin, Berlin, Germany,; 6 Department of Clinical Microbiology, Kwame Nkrumah University of Science and Technology, Kumasi, Ghana

**Keywords:** Human coronaviruses, HCoVs, Ghana, SARS-CoV-2, immunofluorescent assay

## Abstract

**Introduction:**

acute respiratory tract infections (ARIs) are responsible for significant proportions of illnesses and deaths annually. Most of ARIs are of viral etiology, with human coronaviruses (HCoVs) playing a key role. This study was conducted prior to the outbreak of severe acute respiratory syndrome coronavirus-2 (SARS-CoV-2) to provide evidence about the sero-epidemiology of HCoVs in rural areas of Ghana.

**Methods:**

this was a cross-sectional study conducted as part of a large epidemiological study investigating the occurrence of respiratory viruses in 3 rural areas of Ghana; Buoyem, Kwamang and Forikrom. Serum samples were collected and tested for the presence of IgG-antibodies to three HCoVs; HCoV-229E, HCoV-OC43 and HCoV-NL63 using immunofluorescence assay.

**Results:**

of 201 subjects enrolled into the study, 97 (48.3%) were positive for all three viruses. The most prevalent virus was HCoV-229E (23%; 95% CI: 17.2 - 29.3), followed by HCoV-OC43 (17%; 95% CI: 12.4 - 23.4), then HCoV-NL63 (8%, 95% CI: 4.6 - 12.6). Subjects in Kwamang had the highest sero-prevalence for HCoV-NL63 (68.8%). human coronaviruses-229E (41.3%) and HCoV-OC43 (45.7%) were much higher in Forikrom compared to the other study areas. There was however no statistical difference between place of origin and HCoVs positivity. Although blood group O+ and B+ were most common among the recruited subjects, there was no significant association (p = 0.163) between blood group and HCoV infection.

**Conclusion:**

this study reports a 48.3% sero-prevalence of HCoVs (OC43, NL63 and 229E) among rural communities in Ghana. The findings provide useful baseline data that could inform further sero-epidemiological studies on SARS-CoV-2 in Africa.

## Introduction

Acute respiratory tract infections (ARIs) are the leading cause of morbidity and mortality among young children and adults in developing countries [1,2]. A review by Gessner (2011), showed that the highest countries mostly affected by ARIs still remain in Africa [3]. Majority of ARIs are known to be of viral origin with the predominant viruses being respiratory syncytial virus (RSV), influenza virus, rhinoviruses, parainfluenza viruses, human metapneumovirus and human coronaviruses (HCoVs) [4-7]. The role of HCoVs in causing respiratory diseases was however thought to be mild until the outbreak of severe acute respiratory syndrome (SARS) and middle east respiratory syndrome coronavirus (MERS-CoV) which resulted in causing mortality of significant proportions of individuals [8,9]. The world is currently experiencing a worst form of a new strain of coronavirus (SARS-CoV-2) which has so far (as of 16^th^ September, 2020) infected 29,624,865 individuals and resulted in over 930,000 deaths [10]. Because human coronaviruses (HCoV-229E, HCoV-OC43, HCoV-NL63, HCoV-HKU1) are closest to SARS-CoV-2 in terms of its transmissibility, it is thought that immune responses to human coronaviruses could explain the level of disease severity in some populations. Some scientists have hypothesised that low mortality rate of COVID-19 disease in Africa could be due to prior infection with other forms of human coronaviruses [11-14]. However, there is limited sero-epidemiological data to back this assertion in Africa. This study was conducted in three rural communities prior to the outbreak of SARS-CoV-2. The essence was to provide evidence about the sero-epidemiology of human coronaviruses in rural areas of Ghana.

## Methods

**Figure 1:**
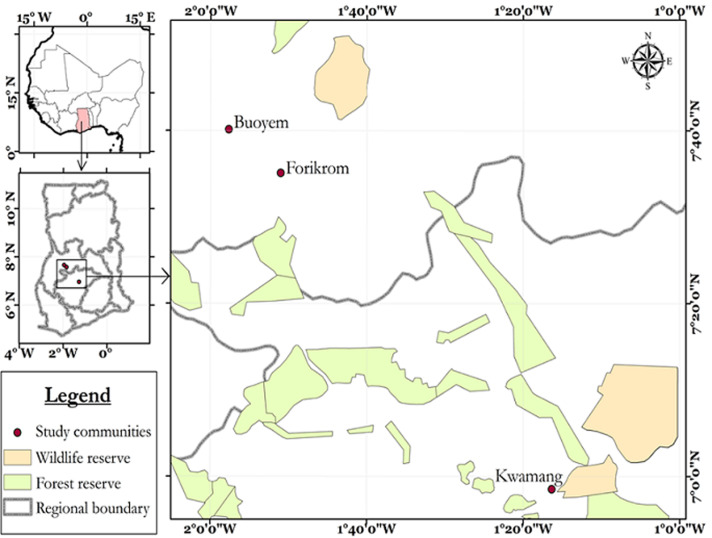
geographical locations of study areas in Ghana

**Study areas:** the study was performed in three rural areas: Buoyem, Kwamang and Forikrom communities. Buoyem and Forikrom are located in the Techiman municipality of the Bono East Region of Ghana. The municipality has a total land surface of 669.7 square kilometers with climate and vegetation that promote the production of food. Kwamang community is in the Sekyere central district of the Ashanti Region (Figure 1). The three communities were purposively selected as part of a large study that was investigating the molecular prevalence of coronaviruses in bats. The three communities have large bat habitats and we hypothesized that persons living in those communities could be exposed to coronaviruses. Studying this population provides sero-epidemiological information in rural communities living near bat habitats.

**Study design:** this was a household-based cross-sectional study that was conducted as part of a large epidemiological study investigating the occurrence of human coronaviruses and other respiratory viruses in Ghana [15]. The study was conducted from September 2011 to September 2012. All subjects were recruited from households if they are above 10 years of age. A purposive sampling technique was adopted to recruit subjects into the study. A subject was selected if he or she had symptoms of upper respiratory illness i.e. sudden onset of any of the following: cough, sneezing, runny nose and nasal congestion. Selection of subjects was done using a cluster sampling design. In Buoyem, the community was divided into four quadrants and clusters of house compounds were marked using satellite images. Houses close to social centres were identified in each cluster and subjects were enrolled from every other household using systematic random sampling design. Researchers approached each household and those who had signs and symptoms of respiratory illness and consented to the study were enrolled. Once subjects became familiar with the study team, radio announcements were made in all communities and eligible subjects were recruited at designated social centres. In Kwamang and Oforikrom communities, satellite images could not be retrieved so major roads that divide the town into four quadrants were identified and social centres at each quadrant of the roads were selected at random. Every other adjacent house starting from the identified social centre was marked and selected and subjects who satisfied the study inclusion criteria were enrolled.

**Ethical approval:** the study protocol was approved by the committee for human research, publications and ethics (CHRPE) of the school of medicine and dentistry, Kwame Nkrumah University of Science and Technology, Kumasi (approval number: CHRPE4 49/09). By means of signature or thumbprinting, written informed consent was obtained for data and sample collection for all subjects. For subjects less than 18 years of age, informed consent was obtained from parents/guardians and assent was obtained from the minors.

**Data collection and statistical analysis:** data such as age, gender, blood group and place of origin were collected from the study subjects and entered into Microsoft excel spreadsheet. Data were later exported to STATA version 12 (Stata Corp, USA) for analysis. Descriptive statistics was used to summarize the distribution of various variables into tables. Proportions (percentages) were computed for categorical variables. Categorical variables and their association with HCoVs were analysed using the Fischer´s exact test or Chi square test where necessary, and alpha level, p<0.05 was considered to be statistically significant. Continuous variables were expressed as medians with their inter-quartile ranges (IQR).

**Sample size:** the sample size for the cross-sectional study was determined based on an estimated HCoV serum IgG antibody prevalence of 90% in the U.S [16]. We assumed a marginal error of 5%, and design effect of 1 which resulted in a minimum sample size of 138. We enrolled exceeded this number in our recruitments in order to increase the power of our analysis. The formula for calculation is shown below: - Let “n” represent the number of subjects needed for the cross-sectional study:

$$\text{n=}\frac{{\left(\text{Z1-}\alpha /2\right)}^{2}\text{ * P * Q * e}}{{\left(\text{d}\right)}^{2}}$$

P = estimated sero-prevalence of HCoVs = 0.90; Q = 1 - P= 0.10; d = estimated marginal error = 0.05; e = estimated design effect = 1; Z_1_-α/2 = 1.96 = value of standard normal distribution corresponding to a significance level of 0.05 for a two-sided test.

**Laboratory methods:** five millilitres (5 ml) of blood samples were taken from each subject who consented to the study into 6 ml gel and clot activator tubes. The blood samples were spun at high speed (13,000 revolutions per minute) for 5 minutes to obtain serum. The serum samples were aliquoted into 5 ml cryotubes and transported in liquid nitrogen to the Bonn Institute of Virology, Germany, for serological analysis using immunofluorescence assay (IFA). Serum samples collected from subjects enrolled were tested for the presence of IgG antibodies to three HCoVs; HCoV-229E, HCoV-OC43 and HCoV-NL63 using IFA. The IFA was performed by applying serum samples to slides prepared in-house. The in-house slides were prepared by expressing spike proteins using VeroB4 cells with plasmids. To test the performance of the IFA, serum samples of three patients who had tested positive for HCoV-NL63, HCoV-OC43 and HCoV-229E were diluted in Euroimmun buffer (Baker, U.S.A) at dilutions of 1: 40, 1: 80, 1: 160, 1: 320, 1: 640 and 1: 1280. Similarly, the serum samples of four rabbits (two not immunized and two immunized) were also diluted with Euroimmun buffer at a dilution of 1: 40 for each serum. The immunized rabbits were used as positive controls for HCoV-229E, HCoV-OC43 and HCoV-NL63. Twenty-five (25μl) of all diluted sera were applied to each well on the glass slide and incubated for 1 hour in a humid box at 37°C. Afterwards, slides were washed three times with phosphate buffered saline in 0.1% tween (PBS-T) for 5 minutes and 25μl of secondary antibodies were applied and incubated for another 30 minutes. The secondary antibodies were goat-anti human Cy2 (Dianova, Germany) diluted to 1: 400 in albuminazid and donkey anti-rabbit Cy2 (Dianova, Germany) diluted to 1: 200. Slides were washed afterwards three times at 5 minutes interval with PBS-T. Cotton-tipped swabs were used to drain off excess liquid outside the stained areas and a drop of mounting medium (DAPI Prolong, Invitrogen, Germany) was applied to the stained wells. The slides were kept in a dark cool environment for 24 hours prior to examination with immunofluorescence microscope.

**Figure 2:**
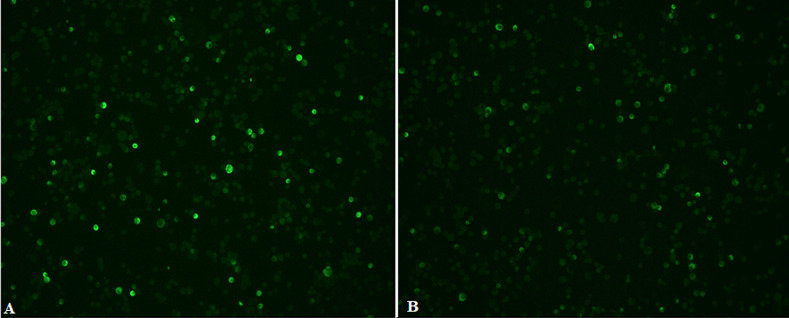
HCoV controls examined with X10 objective lens; images A and B show the positive controls (1:40 dilution) stained for immunofluorescence antibodies against patients infected with HCoV-OC43 and rabbit infected HCoV-NL63, respectively

**Figure 3:**
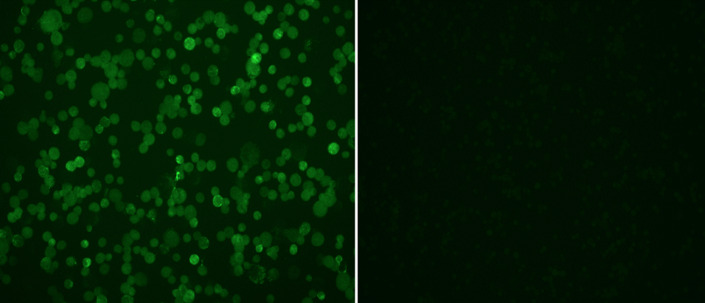
HCoV controls examined with X10 objective lens; A) the positive control (1:40 dilution) stained for immunofluorescence antibodies against patients infected with HCoV-229E; B) image of a negative control from rabbit not immunized against HCoVs

**Figure 4:**
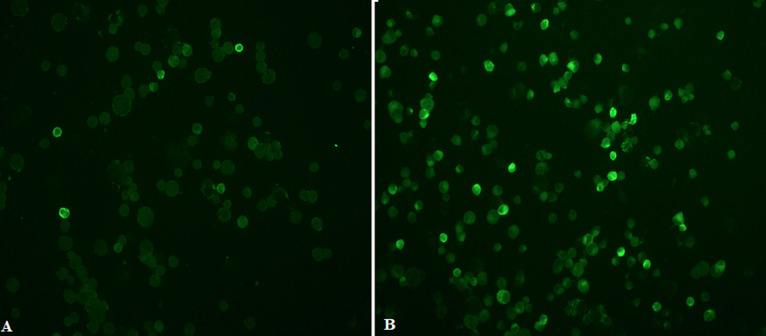
HCoV samples examined with X20 objective lens; images A and B show examples of positive images (1:40 dilution) stained for immunofluorescence antibodies against patients infected with HCoV-OC43 and HCoV-229E, respectively

**Figure 5:**
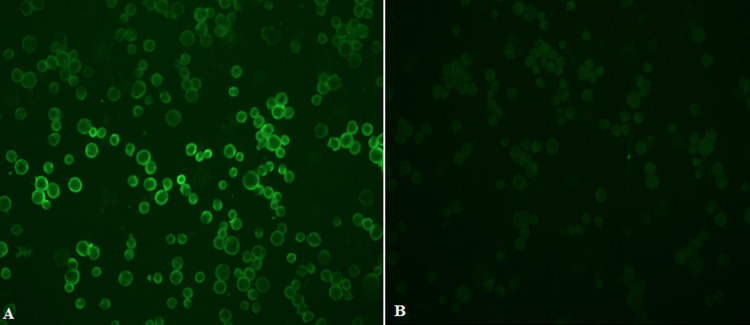
HCoV samples examined with X20 objective lens; image A shows an example of positive image (1:40 dilution) stained for immunofluorescence antibodies against patients infected with HCoV-NL63; B) image of a patient negative for all three viruses

**Examination of stained slides:** slides were examined with an immunofluorescent microscope (Carl Zeiss, Germany) under X10 objective and then confirmed with X20 objective. Pictures were taken with AxioVision Rel 4.8 software. Figure 2(A, B) and Figure 3(A, B) show examples of slide images of the assay controls. Figure 4(A, B) and Figure 5(A, B) show examples of patient slides positive for the three viruses and a negative slide.

## Results

**Sero-prevalence and factors associated with HCoVs:** a total of 201 subjects were enrolled in the serological study. Subjects were tested for IgG antibodies to three HCoVs namely; HCoV-NL63, HCoV-OC43 and HCoV-229E. Of the 201 subjects, 97 (48.3%) were positive for all viruses. The most prevalent virus was HCoV-229E (23%; 95% CI: 17.2 - 29.3), followed by HCoV-OC43 (17%; 95% CI: 12.4 - 23.4), then HCoV-NL63 (8%, 95% CI: 4.6 - 12.6).

**Factors associated with HCoVs infection:** association between some demographic factors and sero-positivity of HCoVs were determined as shown in Table 1. Of all positive HCoV-NL63 subjects, those in Kwamang had the highest sero-prevalence (68.8%). In contrast, HCoV-229E (41.3%) and HCoV-OC43 (45.7%) were much higher in Forikrom compared to the other study areas. There was however no statistical difference between living in any of the study areas and being positive for HCoVs. The median ages of those positive for HCoV-OC43 (47 years, IQR = 33 - 52.5) and HCoV-229E (40 year, IQR = 27 - 54) were higher than negative subjects. The age difference for HCoV-NL63 subjects were similar (p = 0.994). Although blood group O rhesus D positive and B rhesus D positive were most common among the recruited subjects, comparison of the blood group types between subjects positive for HCoVs and those negative showed no significant statistical difference (p = 0.163).

**Table 1:** association between demographic factors and HCoVs sero-positivity

		HCoV-NL63			HCoV-OC43			HCoV-229E	
	Negative	Positive	P-value	Negative	Positive	P-value	Negative	Positive	P-value
	n = 185	n = 16		n = 166	n = 35		n = 155	n = 46	
**Community**			0.08			0.113			0.238
Buoyem n (%)	49 (26.5)	3 (18.8)		46 (27.7)	6 (17.1)		41 (26.5)	11 (23.9)	
Forikrom n (%)	61 (33)	2 (12.5)		47 (28.3)	16 (45.7)		44 (28.4)	19 (41.3)	
Kwamang n (%)	75 (40.5)	11 (68.8)		73 (44)	13 (37.1)		70 (45.2)	16 (34.8)	
**Age median (IQR)**	35 (21.5-52)	33 (19-50)	0.994	30 (19-46.2)	47 (33-52.5)	**0.005**	30 (19-47.5)	40 (27-54)	**0.014**
**Gender n (%)**			0.386			0.195			0.447
Females n (%)	105 (58.3)	7 (43.8)		97 (59.5)	15 (45.5)		89 (58.9)	23 (51.1)	
Males n (%)	75 (41.7)	9 (56.2)		66 (40.5)	18 (54.5)		62 (41.1)	22 (48.9)	
**Blood Group**			0.65			0.711			0.163
A Rh “D” Negative n (%)	1 (0.6)	0 (0)		1 (0.7)	0 (0)		0 (0)	1 (2.3)	
A Rh “D” Positive n (%)	31 (19)	3 (21.4)		25 (17.1)	9 (0)		27 (20.3)	7 (15.9)	
B Rh “D” Positive n (%)	53 (32.5)	5 (35.7)		48 (32.9)	10 (32.3)		47 (35.3)	11 (25)	
B Rh “D” Negative n (%)	1 (0.6)	0 (0)		1 (0.7)	0 (0)		1 (0.8)	0 (0)	
AB Rh “D” Positive n (%)	3 (1.8)	1 (7.1)		4 (2.7)	0 (0)		4 (3)	0 (0)	
AB Rh “D” Negative n (%)	0	0		0	0		0	0	
O Rh “D” Positive n (%)	68 (41.7)	5 (35.7)		62 (42.5)	11 (35.5)		51 (38.3)	22 (50)	
O Rh “D” Negative n (%)	6 (3.7)	0 (0)		5 (3.4)	1 (3.2)		3 (2.3)	3 (6.8)	

## Discussion

The sero-prevalence of HCoVs has not been studied widely in the field of virology. Data on the sero-prevalence and factors associated is limited in Africa. This study identified HCoV-229E as the most prevalent virus, followed by HCoV-OC43. Other studies from developed countries have also reported the detection of IgG antibodies to human coronaviruses in their study subjects [17,18]. The prevalences of HCoVs identified in the above studies were however higher compared to the present study. Severance *et al*. [16] reported over 90% prevalence for each of HCoV-229E, HCoV-OC43 and HCoV-NL63 among U.S.A metropolitan population. Dijkman *et al*. [18] similarly reported over 60% prevalences each for HCoV-229E and HCoV-NL63. The difference in the sero-prevalences could be due to the type of assays used for testing these viruses. The assays used by Dijkman and Severance were based on the nucleocapsid protein and these are known to have conserved regions that could elicit cross-reactivity [19]. Other studies based on the use of spike proteins and whole viruses have reported HCoV prevalences of between 3 and 22% and thus similar to the findings of this study [17,20,21]. Demographic variable analysis revealed age to be associated with HCoV-OC43 and HCoV-229E exposure. The median ages were higher for subjects sero-positive for HCoV-229E and HCoV-OC43 compared to negative subjects. This was to be expected because older subjects might have had prior exposure to HCoV antigens thereby aiding in the development of IgG antibodies. Callow [22] reported humoral immune protection against human coronaviruses. In Africa, especially the sub-Saharan region, there is continuous contact between bats, livestock and humans which might result in exposure to HCoVs and lead to subsequent development of humoral cross-reactivity [23]. Some ABO blood groups are believed to act as receptors for viral transmission. Viruses such as Chikungunya and hepatitis B are reported to be associated with blood groups rhesus positive AB and A [24,25]. Individuals with blood group O were also reported to be protected from SARS-CoV [26]. Guillon *et al*. explained that naturally occurring antibodies (Anti-A) in group O individuals could block the spike proteins of the SARS-CoVs from infecting the epithelial cells lining the small intestines [27]. Similar observations have been made for SARS-CoV-2 [28]. The present study did not find any association between the ABO blood groups and human coronavirus infection. This could be due to the low numbers of study subjects involved in the serology study. Future cross-sectional studies with large numbers are recommended to elucidate these findings.

## Conclusion

Our study has demonstrated a 48.3% sero-prevalence of HCoVs (OC43, NL63 and 229E) among individuals in Ghana. The findings provide a baseline information that could enhance our knowledge about the behaviour of SARS-CoV-2 and also inform further sero-epidemiological studies on this new virus in Africa.

### What is known about this topic

- Acute respiratory tract infections are predominantly caused by viruses including human coronaviruses;
- Human coronaviruses are closest to the new coronavirus strain (SARS-CoV-2).

### What this study adds

- There is high sero-prevalence of human coronaviruses (HCoVs) in rural Ghana;
- This study provides fundamental information about HCoVs which is useful for future sero-epidemiological studies in Africa.
